# Supplementary material for: SERS nose arrays based on a signal differentiation approach for TNT gas detection
Source: Commun Chem. 2025 Aug 18;8:250. doi: 10.1038/s42004-025-01656-2 (PMC12361517; doi:10.1038/s42004-025-01656-2)
Supplement: Supplementary file 2 — Supplementary Information [file 42004_2025_1656_MOESM2_ESM.pdf]

Supplementary Information

SERS nose arrays based on a signal differentiation approach for TNT gas detection

Peitao Dong[1,#], Haiyang Yang[2,#], Tianran Wang[3,1]✉, Siyue Xiong[1], Li Kuang[2], Weihong Qi[4], Xiaohua Chen[5], Lixia Yang[6], Qiuyun Fan[7], Dingbang Xiao[1] & Xuezhong Wu[1]

[1] College of Intelligence Science and Technology, National University of Defense Technology, Changsha, Hunan, China.

[2] School of Computer Science and Engineering, Central South University, Changsha, Hunan, China.

[3] School of Intelligent Manufacturing and Electronic Engineering, Wenzhou University of Technology, Wenzhou, Zhejiang, China.

[4] State Key Laboratory of Solidification Processing and Center of Advanced Lubrication and Seal Materials, Northwestern Polytechnical University, Xi'an, China.

[5] Department of Laboratory Medicine, General Hospital of Central Theater Command, Wuhan, Hubei, China.

[6] Changsha Institute for Food and Drug Control, Changsha, Hunan, China.

[7] Hunan Changsha Ecological and Environmental Monitoring Center, Changsha, Hunan, China.

#These authors contributed equally: Peitao Dong and Haiyang Yang.

✉ Author to whom correspondence should be addressed: Tianran Wang, e-mail: wangtianran@wzut.edu.cn

Table of contents for supporting information of figures

Supplementary Fig. 1s| Tip-to-tip hotspots for a.Au nanostars, b. Au nanorods and c. Au nanobipyramid of nanostructures.

Supplementary Fig. 2s| The electromagnetic field distributions at the tip-to-tip regions of a.Au nanostars, b. Au nanorods, and c. Au nanobipyramid.

Supplementary Fig. 3s| a The transmission electron microscopy (TEM) image of Au seeds. b Particle size distribution diagram of Au seeds.

Supplementary Fig. 4s| The transmission electron microscopy images of AuNBPs prepared from gold seed solutions with concentrations of a 70μL, b 90μL, c 110μL, and d 130μL.

Supplementary Fig. 5s| The absorption spectra of AuNBPs synthesized using gold seed solution volumes of 70 μL, 90 μL, 110 μL, and 130 μL.

Supplementary Fig. 6s| The Zeta Distribution Data of a Ti_3C_2 MXene, b Mo2C MXene, and c AuNBPs.

Supplementary Fig. 7s| a-d. The TEM and e. the SEM images of the heterostructure at different magnifications

Supplementary Fig. 8s| The electromagnetic field intensity generated under excitation light perpendicular to the head-to-head contact plane of two AuNBPs, the corresponding E/E_0 maximum value is 1.12.

Supplementary Fig. 9s| Optimized structural diagrams of the four adsorption models.

Supplementary Fig. 10s| The intrinsic Raman spectra of TNT, 4-NT, and 2,4-DNPA powder samples.

Supplementary Fig. 11s| Raman spectra of Mo_2C MXene/AuNBPs composite SERS substrates modified with 10^{-5} M and 10^{-6} M capturers before (b and d) and after (a and c) detecting 15 ppb TNT.

Supplementary Fig. 12s| (i) Raman spectra of the Mo_2C MXene-AuNBPs-PATP after adsorbing TNT for different durations (0 min, 10 min, 20 min, 30 min, 40 min, 50 min, 60 min) and (ii) the intensity plots of the Raman characteristic peaks of TNT at different adsorption times.

Supplementary Fig. 13s| Raman intensity distribution at 1350 cm^{-1} for 75 random points.

Supplementary Fig. 14s| SERS spectra for detecting TNT gas at different concentrations (932 ppb, 260 ppb, 67.1 ppb, 15 ppb, 7.4 ppb) based on the Mo_2C MXene-AuNBPs-PATP substrate.

Supplementary Fig. 15s| Detection of Raman spectra for TNT, 4-NT, and 2,4-DNPA gases

molecules at 60°C using Mo_2C MXene-AuNBPs-PATP.

Supplementary Fig. 16s| Schematic diagram of the charge transfer mechanism between 2,4-DNPA and MXenes.

Supplementary Fig. 17s| The RF confusion matrix for the classification of TNT (label 0) and 2,4-DNPA (label 1) gases at 30℃ using different models.

Supplementary Fig. 18s| a. The RF classification performance of TNT and 2,4-DNPA gases at 30℃ using different models and b. the classification accuracy results of the different ML methods for the prediction set of TNT and 2,4-DNPA gases at 30℃.

Supplementary Fig. 19s| a. The RF confusion matrix for the classification of 7.4ppb (label 0) and 15ppb (label 1) of TNT gases using different models. b (i) The RF classification performance of 7.4ppb and 15ppb TNT using RF different models. (ii) The classification accuracy results of the different ML methods for the prediction set of 7.4ppb and 15ppb of TNT gas.

Table of contents for supporting information of tables

Supplementary Table 1s The peak and area information for the zeta potential of Ti_3C_2 Mxene

Supplementary Table 2s The peak and area information for the zeta potential of Mo_2C MXene

Supplementary Table 3s The peak and area information for the zeta potential of AuNBPs

Supplementary Table 4s The molar fraction of the saturated vapor pressure of TNT gas at different temperatures.

Supplementary Table 5s Classification Validation Results of TNT and 2,4-DNPA gases at 25℃ by 10-fold cross-validation using different machine learning algorithms by inputting information of different unit models.

Supplementary Table 6s Classification Validation Results for 15ppb and 7.4ppb TNT by 10-fold cross-validation using different machine learning algorithms by inputting information of different unit models.

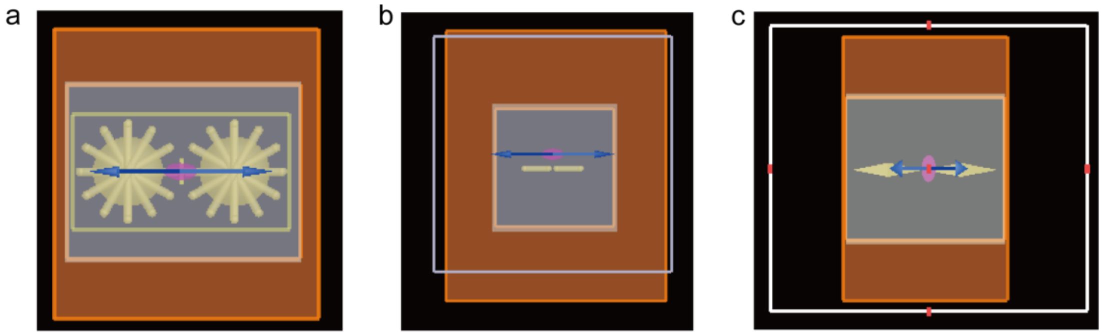

Supplementary Fig. 1s| Tip-to-tip hotspots for a.Au nanostars, b. Au nanorods and c. Au nanobipyramid of nanostructures.

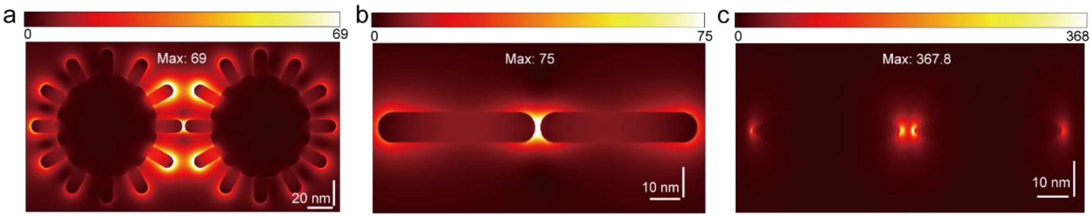

Supplementary Fig. 2s| The electromagnetic field distributions at the tip-to-tip regions of a.Au nanostars, b. Au nanorods, and c. Au nanobipyramid.

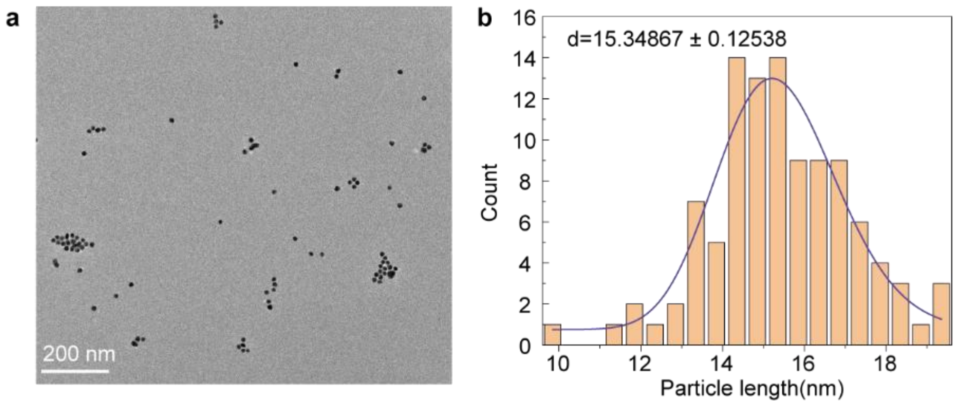

Supplementary Fig. 3s| a The transmission electron microscopy (TEM) image of Au seeds. b Particle size distribution diagram of Au seeds.

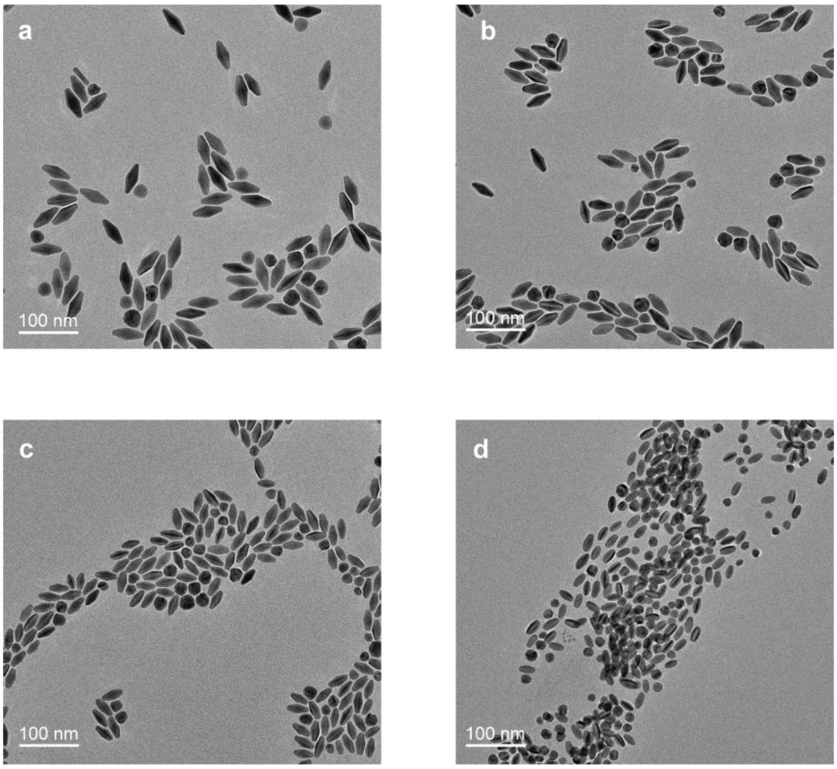

Supplementary Fig. 4s| The transmission electron microscopy images of AuNBPs prepared from gold seed solutions with concentrations of a 70μL, b 90μL, c 110μL, and d 130μL.

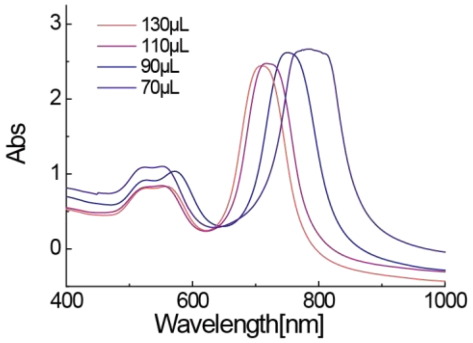

Supplementary Fig. 5s| The absorption spectra of AuNBPs synthesized using gold seed solution volumes of 70 μL, 90 μL, 110 μL, and 130 μL.

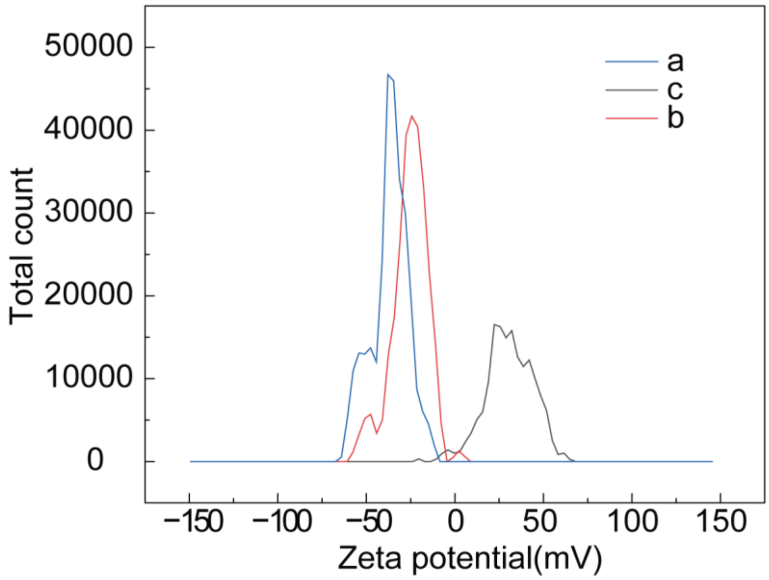

Supplementary Fig. 6s| The Zeta Distribution Data of a Ti_3C_2 MXene, b Mo_2C MXene, and c AuNBPs.

Supplementary Table 1s The peak and area information for the zeta potential of Ti_3C_2 MXene

	Mean(mV)	Area(%)	St Dev(mV)
Peak 1	-32.8	74.1	6.97
Peak 2	-55.0	13.6	3.44
Peak 3	-47.8	12.3	2.63

Supplementary Table 2s The peak and area information for the zeta potential of Mo_2C MXene

	Mean(mV)	Area(%)	St Dev(mV)
Peak 1	-24.1	92.5	7.89
Peak 2	-49.6	6.6	3.81
Peak 3	2.4	0.8	2.25

Supplementary Table 3s The peak and area information for the zeta potential of AuNBPs

	Mean(mV)	Area(%)	St Dev(mV)
Peak 1	20.1	40.5	7.00
Peak 2	33.4	29.1	3.59
Peak 3	45.0	27.1	5.15

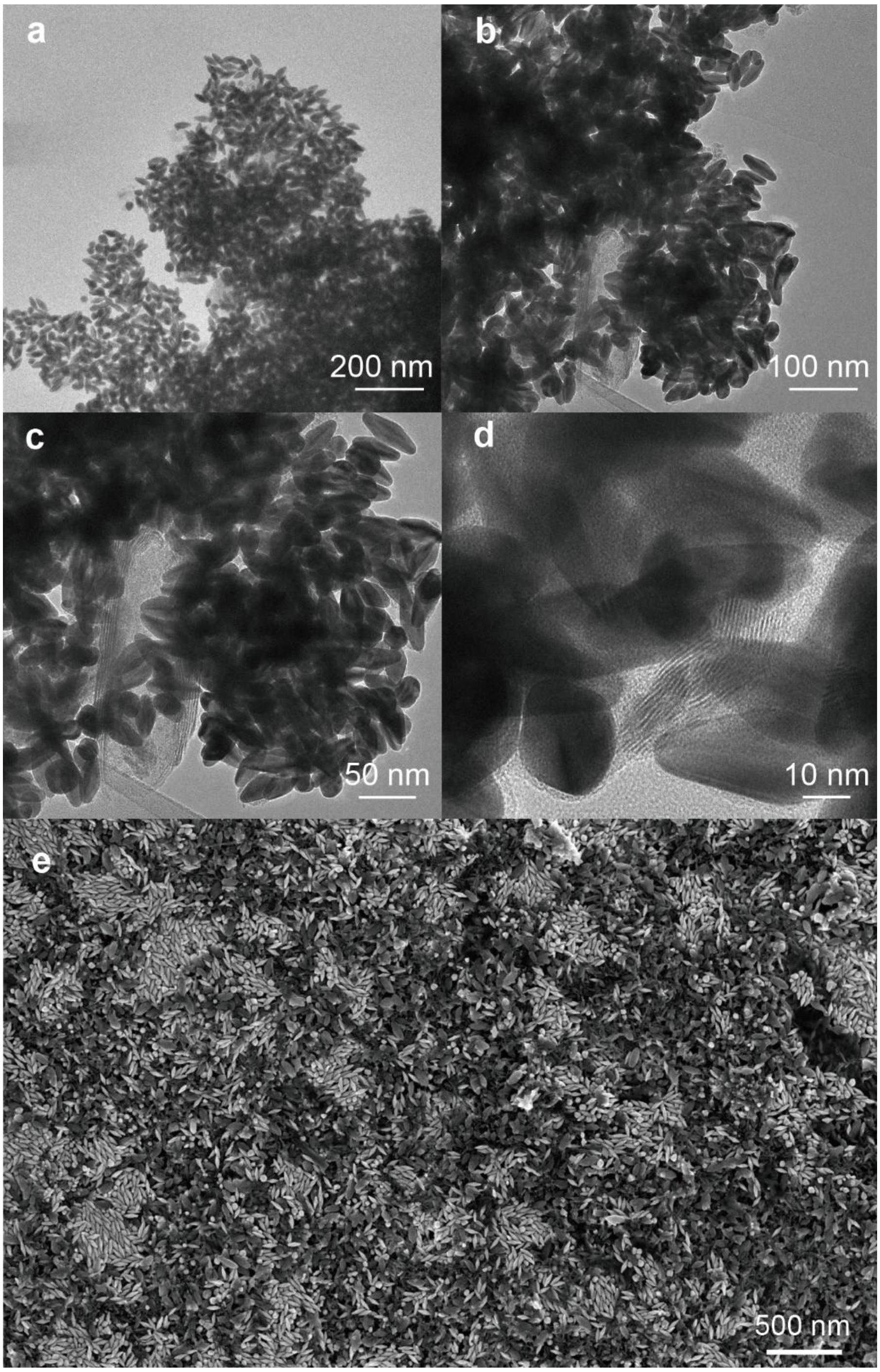

Supplementary Fig. 7s| a-d. The TEM and e. the SEM images of the heterostructure at different magnifications

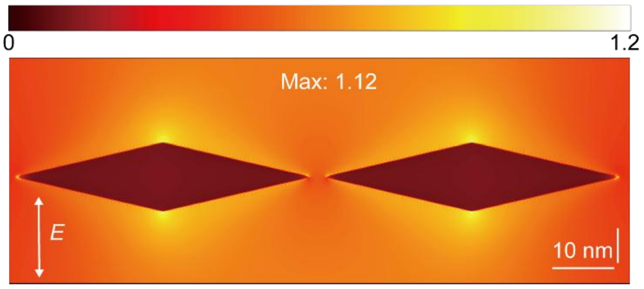

Supplementary Fig. 8s| The electromagnetic field intensity generated under excitation light perpendicular to the head-to-head contact plane of two AuNBPs, the corresponding E/E_0 maximum value is 1.12.

DFT calculation method

The DFT calculations were carried out using the Vienna Ab-initio Simulation Package (VASP)[1,2] with the frozen-core all-electron projector-augment-wave (PAW) [3,4] method. The Perdew-Burke-Ernzerhof (PBE) [5]of generalized gradient approximation (GGA) was adopted to describe the exchange and correlation potential. The cutoff energy for the plane-wave basis set was set to 450 eV. The 3×3×1 Monkhorst-Pack k-point[6] sampling was used. The mono-layer 5×5 Mo_2C and 5×5 Ti_3C_2 supercell were used to investigate the adsorption of 4MBA and PATP. A vacuum region of 25 Å was added above the supercell models to minimize the interactions between neighboring systems. The geometry optimizations were performed until the forces on each ion was reduced below 0.01 eV/Å. The resulting structures were then used to calculate the electronic structures. The adsorption energy (E_{ads}), is calculated using the expression

$$E_{ads} = E_{molecule+surface} - E_{surface} - E_{molecule}$$

where $E_{surface}$ is the energy of Mo_2C and Ti_3C_2 surfaces, $E_{molecule}$ represents the energy of 4MBA and PATP, $E_{molecule+surface}$ represents the total energy of the adsorbed system.

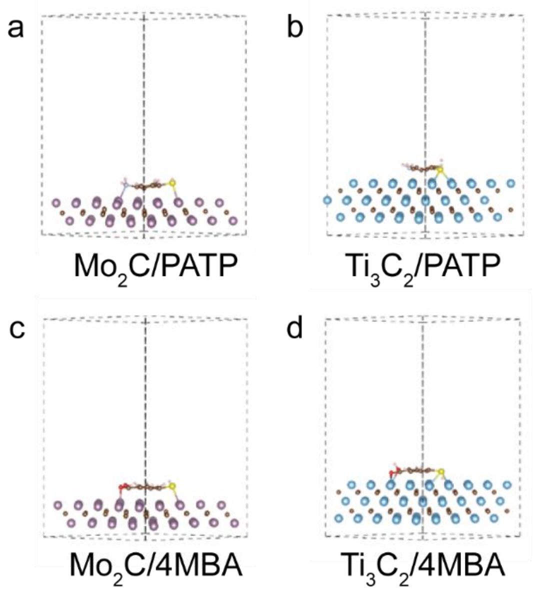

Supplementary Fig. 9s| Optimized structural diagrams of the four adsorption models.

Calculation of TNT Gas Concentration

For TNT molecules, the crystal exhibits varying saturated vapor pressures under the influence of temperature. Based on thermodynamic principles, the saturated vapor pressure of the explosive TNT at different temperatures in air can be studied using the Clausius-Clapeyron equation. The specific formula is as follows[7]:

$$log_{10}p = A - \frac{B}{T} \quad (1)$$

In the equation: p represents the vapor pressure of TNT, with units in Torr. T is the temperature, expressed in Kelvin (K). A and B are constants typically obtained through experiments, depending on the intrinsic properties of the material. B is given as:

$$B = \frac{\Delta H}{2.303R} \quad (2)$$

In the equation, ΔH is the enthalpy of sublimation. R is the gas constant.

Typically, the saturated vapor pressure of a crystal varies within a specific temperature range. According to literature[8], for TNT in the temperature range of 12-80°C, the constants A and B are 14.74 and 5960, respectively. Therefore, equation (1) can be used to estimate the saturated vapor pressure of TNT at different temperatures.The saturated vapor pressure of TNT can then be converted into the molar fraction of TNT vapor using the following formula, with results shown in Table S4:

$$X = \frac{Vapor\ presssure\ of\ TNT}{Total\ pressure} \quad (3)$$

Where: X is the molar fraction of TNT vapor. $Vapor\ presssure\ of\ TNT$ is the saturated vapor pressure of TNT (Torr). $Total\ pressure$ is the total atmospheric pressure (Torr).

Supplementary Table 4s. The molar fraction of the saturated vapor pressure of TNT gas at different temperatures.

Temperature(K)	Concentration (ppb)
298	7.400285194
303	15.81074012
313	67.10792738
323	260.4588341
333	931.8521167

Considering the actual gas generation process, the saturated vapor pressure of TNT at a specific temperature is often limited to a small region near the structure of TNT powders. For the diffusion of TNT gas molecules, there are typically three processes : desorption, adsorption, and detachment. When the distance to the TNT powder is sufficiently small, TNT molecules can generally reach an equilibrium state of adsorption and desorption. Within this region, the process can be described by the free path λ of TNT molecular diffusion[9]. Beyond the range of λ, TNT molecules detach from the surface of the TNT powder crystals and diffuse outward. In this case, the greater the number of detached TNT molecules, the higher the corresponding saturated vapor pressure of TNT molecules in the gas phase.

According to Gershanik's theory[10], when molecular convection and accumulation are neglected, the overall diffusion and transport process of TNT molecules is considered to be in an equilibrium state. At this point, the concentration C of TNT molecules at a height H from the TNT powder surface can be calculated using equation:

$$C = \frac{aC_1(b-H)+bC_2(H-a)}{H(b-a)} \tag{4}$$

In the equation, the molecular concentrations corresponding to a and b are C_1 and C_2, respectively. In this case, as the distance from the TNT solid surface approaches infinity, b approaches infinity, and the molecular concentration C_2 approaches zero. Therefore, above equation can be simplified to:

$$C = \frac{a}{H} C_1 \tag{5}$$

To determine the value of a, we assume that the TNT molecules are within the free path range λ, where the vapor pressure corresponds to the saturated vapor pressure of TNT. By simplifying the above equation, at this condition, λ can be treated as the free path, and C_1 can be considered the saturated vapor pressure C_{sat}^{TNT}. Since the saturated vapor pressure of TNT is temperature-dependent, C_{sat}^{TNT} becomes a constant when the temperature is fixed. Thus, the above equation can be further rewritten as:

$$C^{TNT}(T) = \frac{\lambda}{H} C_{sat}^{TNT}(T) \tag{6}$$

According to equation (6), at a given temperature: H represents the distance from the TNT solid surface, and C_{sat}^{TNT} represents the saturated vapor pressure of TNT at

that temperature. Once $\lambda$is known, the TNT gas concentration at a distance H from the TNT solid surface can be calculated using equation (6).

For the calculation of the free path λ, it can be determined using equation (7) and (8):

$$\lambda = \frac{3D}{v} \tag{7}$$

$$v = \sqrt{\frac{8k_BT}{\pi m}} = \sqrt{\frac{8RT}{\pi M}} \tag{8}$$

In this context, the symbols represent the following: D is diffusion coefficient. v is average thermal velocity of the gas molecules. k_B is Boltzmann constant. m is the mass of the gas molecule. R is universal gas constant. T is thermodynamic temperature (in Kelvin). M is Molar mass of the gas. According to the literature, the reported value of the diffusion coefficient is D=5.59×10^{-6}m^2/s[11]. By calculation, the free path λ 27°C is determined to be 3.17×10^{-6}m. According to eq(5.6), at H of 10 cm above solid TNT, C^{TNT} decreases from 10 ppb (the saturated vapor pressure of TNT at 27°C) to 0.32 ppt. It can be observed that, at a constant temperature, the concentration of TNT gas generated in a sealed environment is typically lower near the bottle opening. To minimize this effect, the gas generation time should be as long as possible during the gas emission process. To avoid other errors, the time for capturing TNT at the bottle opening using a composite SERS substrate should be sufficient to minimize the effect of distance on diffusion. Additionally, it is assumed by default that the concentration of gas at all positions within the bottle corresponds to the saturated vapor pressure concentration during detection.

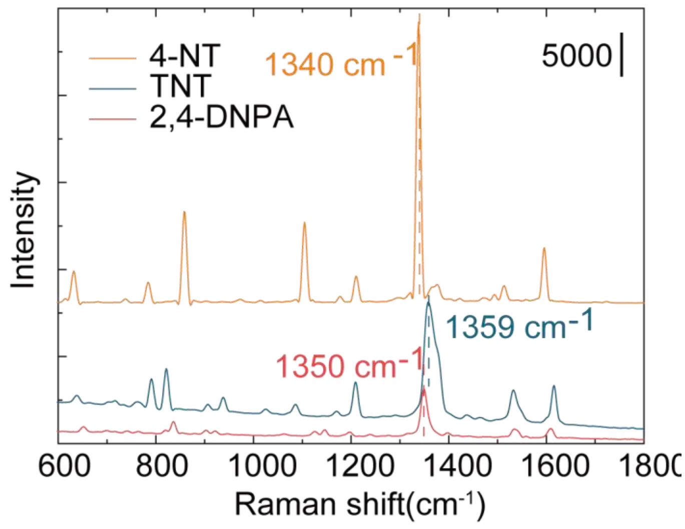

Supplementary Fig. 10s| The intrinsic Raman spectra of TNT, 4-NT, and 2,4-DNPA powder samples.

Calculation of Surface density of capture molecules on MXene-AuNBPs.

Determine the total number of capture molecules: The total amount n of captured molecules can be calculated using Equation 9.

$$n = \frac{V}{C} N_A \quad (9)$$

where V is the volume of the solution, C is the molar concentration of the captured molecules and N_A is Avogadro's number. In our experiment, 40μL captured molecules of 10^{-6}M were used. The total amount n of captured molecules was about 2.41×10^{13}.

Based on the preparation method of MXene-AuNBPs-PATP (4MBA or 6MNA), the mass of 200 μL of MXene (1 mg/mL) is 0.2 mg. Due to the tight connection of AuNBPs on the surface of MXene-AuNBPs, we estimated the surface area of MXene to represent the surface area of MXene-AuNBPs. To simplify the calculation, we used the surface area of MXene instead of calculating the surface area of MXene-AuNBPs. According to [Applied Surface Science 473 (2019) 409–418], the specific surface area of few-layer MXene is 69.11 m^2/g, leading us to estimate the surface area $\boldsymbol{A}$ of MXene-AuNBPs to be approximately 0.0138 m^2.

Surface density σ of capture molecules on MXene-AuNBPs can be determined by Eq 10.

$$\sigma = n/A \quad (10)$$

The surface density σ is 1.739×10^{-3}molecules/nm^2.

The surface area of AuNBPs is also calculated to evaluated the amount of captured molecules on the surface of AuNBPs on MXene-AuNBPs.

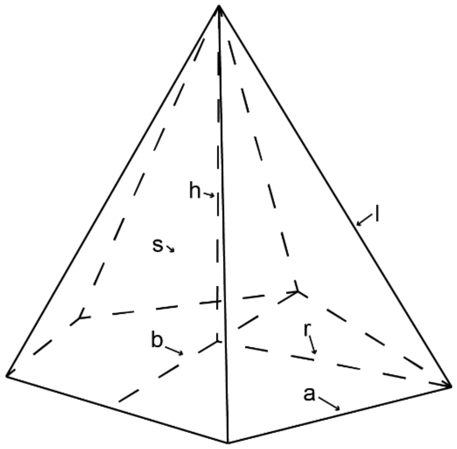

To calculate the surface area of AuNBP, we take half of the AuNBP—the pentagonal pyramid—as an example for the calculation.

Based on the TEM images of the gold nanodumbbells, we can determine that the height *h* is 26.5 nm and the width *b* is 20 nm. The side length *a* can be calculated using the following formula 11:

$$a = \frac{2b}{1+\sqrt{5}} \tag{11}$$

Next, we need to calculate the slant height *l*. The slant height can be calculated using the eq 12:

$$l = \sqrt{h^2 + r^2} \tag{12}$$

where *r* is the circumradius of the pentagonal base. The circumradius of the pentagonal base can be calculated as follows:

$$r = \frac{a}{2\sin(\pi/5)} \tag{13}$$

We can get *a*, *l* and *r* is 15.38nm, 13.76nm and 29.76nm, respectively. Then we can get the surface area s is 229nm^2 by eq 14

$$s = al/2 \tag{14}$$

Since the AuNBP lies flat on the MXene surface, it has two faces in contact with the MXene. Excluding the shadow effect, the exposed surfaces that can interact with adsorbed molecules consist of six faces. Therefore, we estimate the surface area *S* of one AuNBP to be 1374 nm^2 by S=6s, where s represents the area of one face.

Finally, the number of capture molecules that a single AuNBP can bind is approximately 2.4 molecules by $S \times \sigma$.

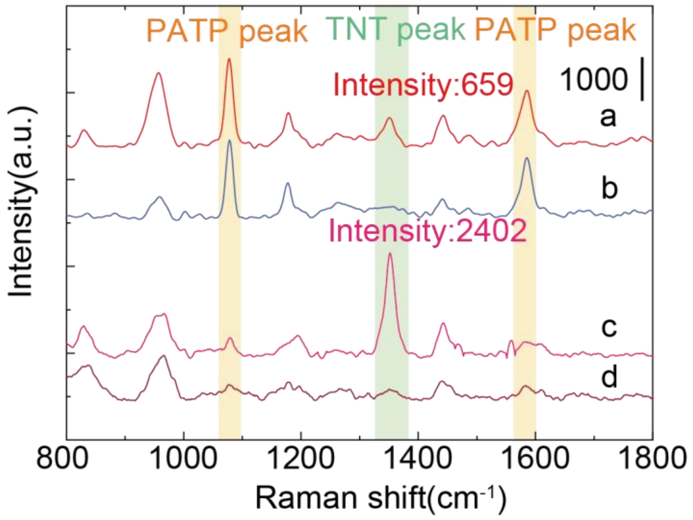

Supplementary Fig. 11s| Raman spectra of Mo_2C MXene/AuNBPs composite SERS substrates modified with 10^{-5} M and 10^{-6} M capturers before (b and d) and after (a and c) detecting 15 ppb TNT.

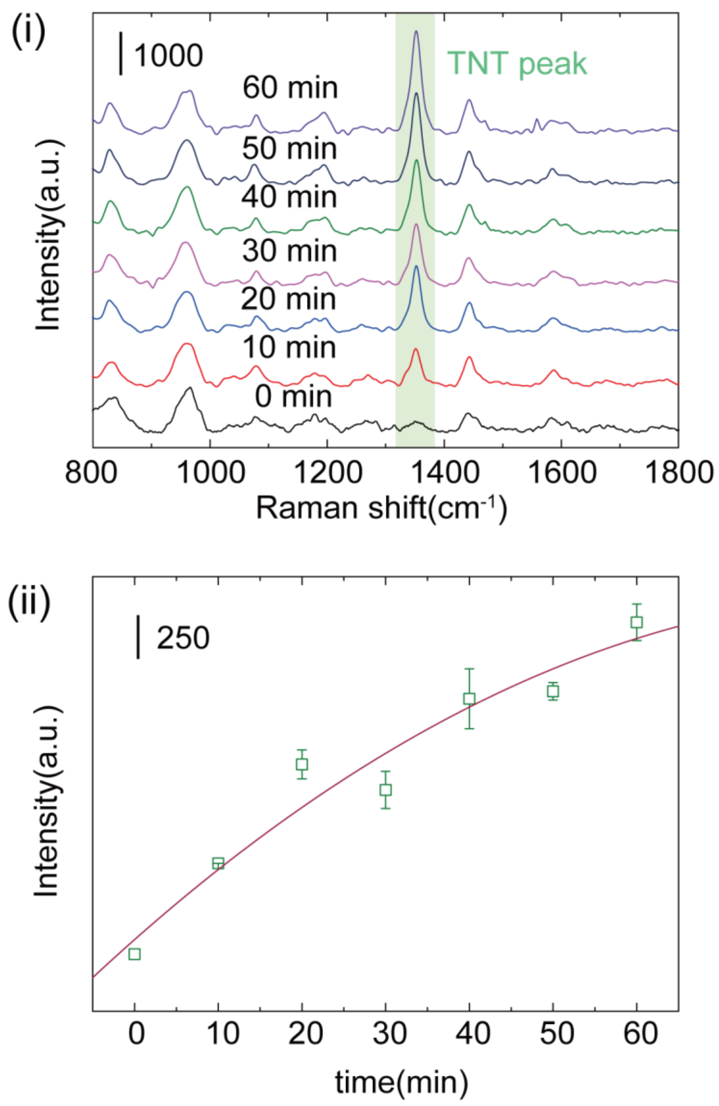

Supplementary Fig. 12s| (i) Raman spectra of the Mo_2C MXene-AuNBPs-PATP after adsorbing TNT for different durations (0 min, 10 min, 20 min, 30 min, 40 min, 50 min, 60 min) and (ii) the intensity plots of the Raman characteristic peaks of TNT at different adsorption times.

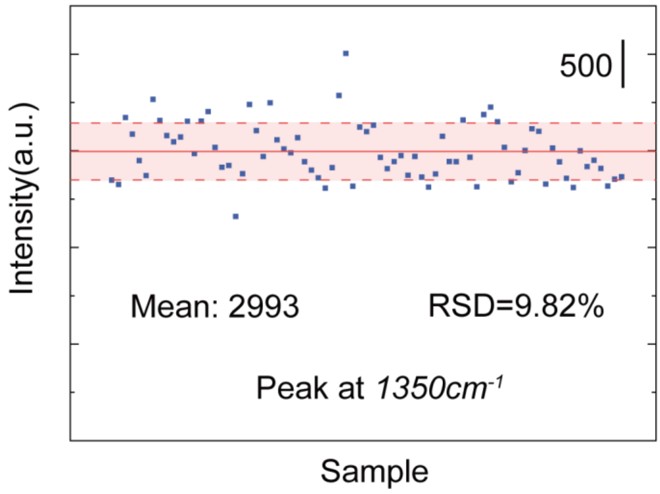

Supplementary Fig. 13s| Raman intensity distribution at 1350 cm^{-1} for 75 random points.

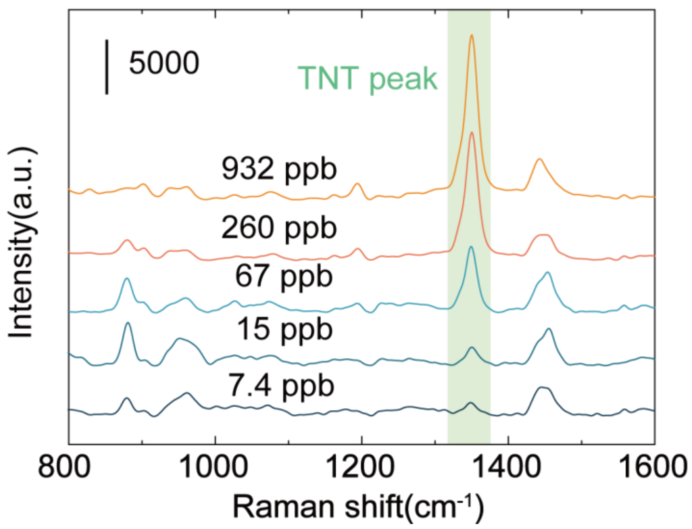

Supplementary Fig. 14s| SERS spectra for detecting TNT gas at different concentrations (932 ppb, 260 ppb, 67.1 ppb, 15 ppb, 7.4 ppb) based on the Mo_2C MXene-AuNBPs-PATP substrate.

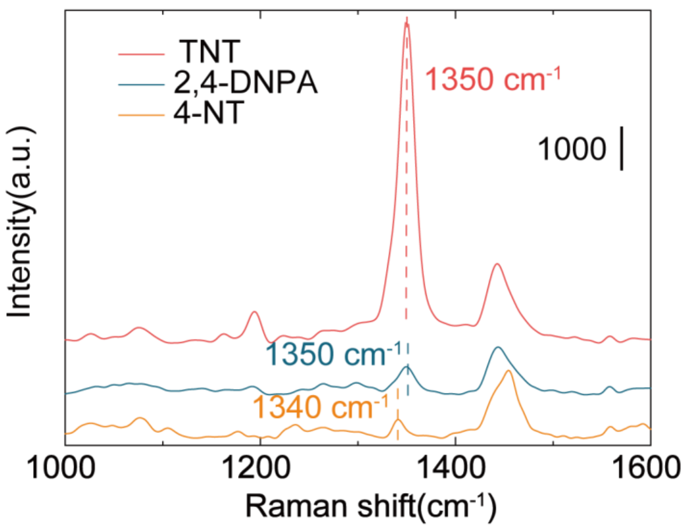

Supplementary Fig. 15s| Detection of Raman spectra for TNT, 4-NT, and 2,4-DNPA gases molecules at 60°C using Mo_2C MXene-AuNBPs-PATP.

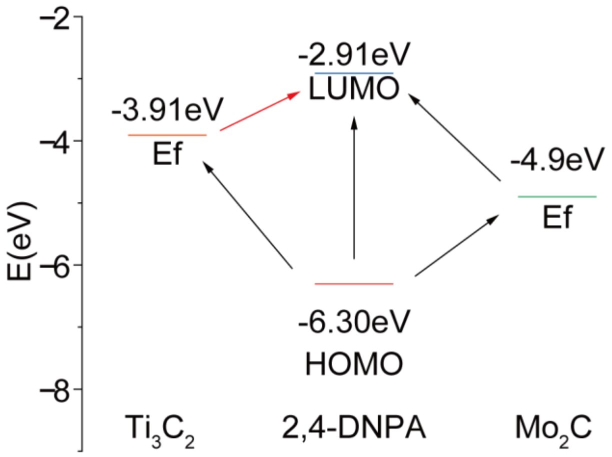

Supplementary Fig. 16s| Schematic diagram of the charge transfer mechanism between 2,4-DNPA and MXenes.

Machine Learning-Assisted Classification and Detection

In this study, a total of 16,200 data entries were collected. For temperature and duration variables, the dataset was split into training and testing sets in an 8:2 ratio. The machine learning model was trained on the training set to learn the relationship between the SERS nasal sensor samples and their labels. The model then predicted labels for the unlabeled testing set to validate classification performance. To ensure experimental rigor and reliability, the experimental results were averaged over 10 random splits of the dataset. This study employed four commonly used evaluation metrics in machine learning classification: precision, recall, accuracy, and F1. We use the following metrics to measure the model performance in mismatch detection. Here:

Precision is the percentage of predicted detection targets that are accurately predicted,

$$\text{Precision} = \frac{TP}{TP + FP}$$

where *TP* is true positive, which refers to the positive samples that are correctly predicted as positive by the model. *FP* is false positive, which refers to the negative samples that are incorrectly predicted as positive by the model.

Recall measures how much TNT gases are accurately predicted,

$$\text{Recall} = \frac{TP}{TP + FN}$$

where *FN* is false negative, which refers to the positive samples that are incorrectly predicted as negative by the model.

F1 combines both of the above two metrics.

$$\text{F1} = \frac{2 * \text{Precision} * \text{Recall}}{\text{Precision} + \text{Recall}}$$

Accuracy is the percentage of overall accurate predictions for both types of samples,

$$\text{Accuracy} = \frac{TP}{TP + TN + FP + FN}$$

where *TN* is true negative, which refers to the negative samples that are correctly predicted as negative by the model.

Supplementary Table 5s Classification Validation Results of TNT and 2,4-DNPA gases at 25℃ by 10-fold cross-validation using different machine learning algorithms by inputting information of different unit models.

ML	models	Accuracy(%)	Precision(%)	Recall(%)	F1(%)
RF	unit a	88.88889	88.25279	88.74638	88.249
	unit b	85	84.64318	85.057	84.23079
	unit c	91.94444	91.67732	91.91484	91.56374
	unit d	96.66667	96.5809	96.60976	96.49176
	unit e	99.16667	98.82751	99.37209	99.06879
	unit f	92.5	92.04781	92.06232	91.97889
	SD-SERS arrays	99.44444	99.33712	99.42308	99.35748
LR	unit a	88.33333	88.46955	88.29619	87.82764
	unit b	88.88889	88.1394	89.07673	88.28313
	unit c	90.83333	90.8939	90.46885	90.26121
	unit d	96.94444	96.73658	97.07878	96.80051
	unit e	100	100	100	100
	unit f	93.33333	92.73275	93.7776	92.97725
	SD-SERS arrays	100	100	100	100
KNN	unit a	87.77778	87.48853	87.4962	86.99393
	unit b	86.11111	86.26474	86.84789	85.63999
	unit c	92.22222	92.03342	92.51241	91.88354
	unit d	96.11111	96.07785	95.94309	95.88863
	unit e	99.44444	99.21212	99.58042	99.37517
	unit f	93.05556	92.43793	92.92963	92.6268
	SD-SERS arrays	100	100	100	100
SVM	unit a	90	89.85703	90.05822	89.42831
	unit b	86.11111	85.98159	86.95196	85.59815
	unit c	91.66667	91.58022	91.5579	91.20024
	unit d	96.94444	96.80337	96.83468	96.69703
	unit e	99.72222	99.54545	99.80769	99.66387
	unit f	91.66667	91.05177	91.71992	91.25965
	SD-SERS arrays	100	100	100	100

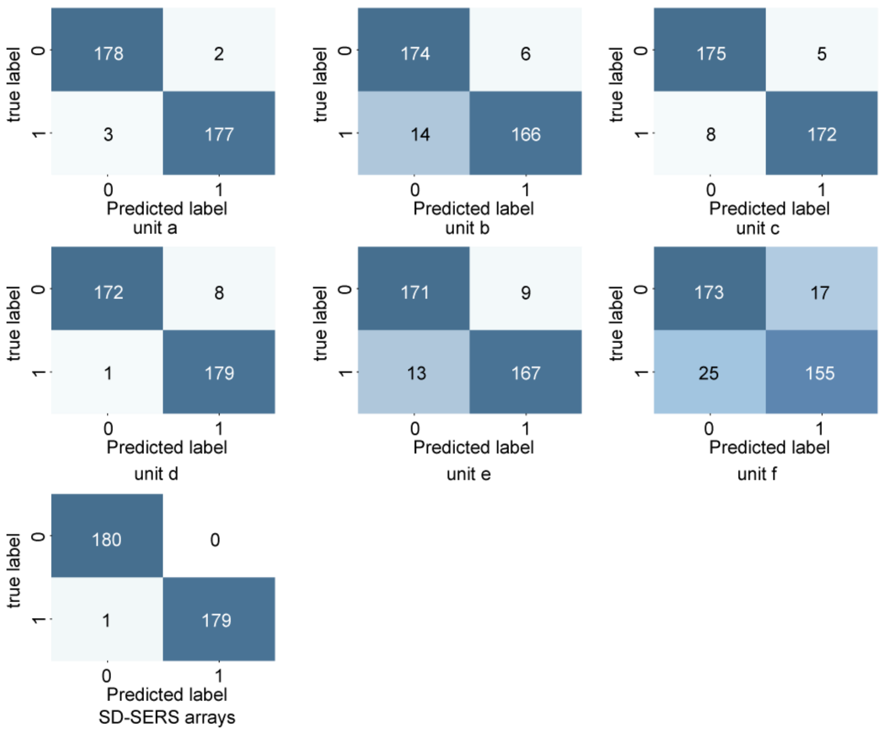

Supplementary Fig. 17s| The RF confusion matrix for the classification of TNT (label 0) and 2,4-DNPA (label 1) gases at 30°C using different models.

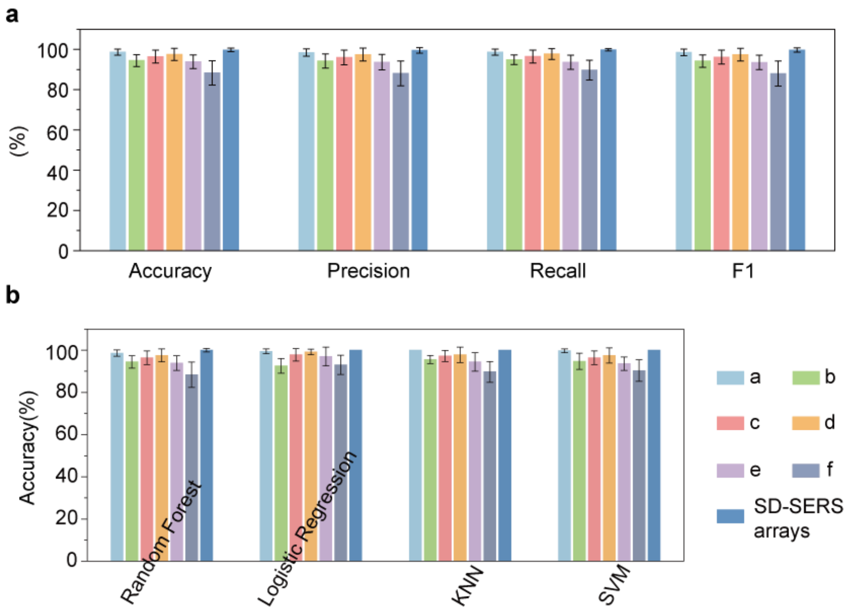

Supplementary Fig. 18s| a. The RF classification performance of TNT and 2,4-DNPA gases at 30℃ using different models and b. the classification accuracy results of the different ML methods for the prediction set of TNT and 2,4-DNPA gases at 30℃.

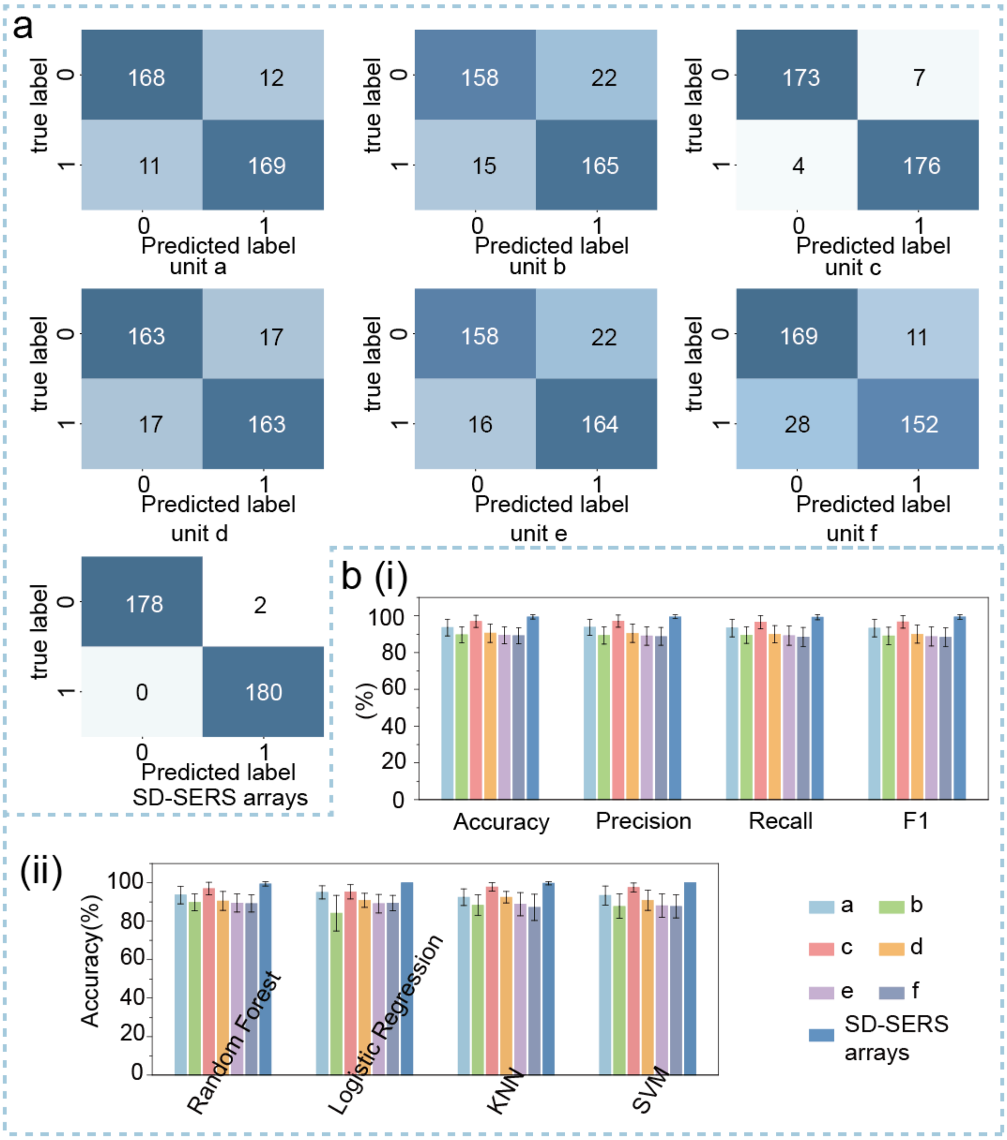

Supplementary Fig. 19s| a. The RF confusion matrix for the classification of 7.4ppb (label 0) and 15ppb (label 1) of TNT gases using different models. b (i) The RF classification performance of 7.4ppb and 15ppb TNT using RF different models. (ii) The classification accuracy results of the different ML methods for the prediction set of 7.4ppb and 15ppb of TNT gas.

Supplementary Table 6s Classification Validation Results for 15ppb and 7.4ppb TNT by 10-fold cross-validation using different machine learning algorithms by inputting information of different unit models.

ML	models	Accuracy(%)	Precision(%)	Recall(%)	F1(%)
RF	unit a	93.61111	93.79016958	93.42837347	93.32941514
	unit b	89.72222	89.33314501	89.46218754	89.08583402
	unit c	96.94444	97.10449735	96.58632684	96.76913935
	unit d	90.55556	90.53980558	90.03154515	90.05465446
	unit e	89.44444	89.06357079	89.23783398	88.77571006
	unit f	89.16667	88.81467897	88.52157041	88.37643991
	SD-SERS arrays	99.44444	99.51388889	99.35222672	99.41562474
LR	unit a	95	94.82999946	94.9171071	94.72958971
	unit b	84.16667	83.98422734	83.75888597	83.4394573
	unit c	95.27778	95.3613413	94.91499606	94.99602286
	unit d	90.83333	90.52436439	90.64516773	90.31323433
	unit e	89.16667	88.70783523	89.08715585	88.57449156
	unit f	89.44444	89.04784314	89.31899165	88.94449845
	SD-SERS arrays	100	100	100	100
KNN	unit a	92.5	92.38717946	92.48878317	92.11016761
	unit b	88.33333	88.17620744	88.35806951	87.72267448
	unit c	97.77778	98.10628614	97.34783579	97.61926483
	unit d	92.5	92.64447967	92.15579677	92.04378276
	unit e	88.88889	88.05609246	88.6896067	88.21901133
	unit f	87.22222	87.10189155	86.41464072	86.32729207
	SD-SERS arrays	99.72222	99.66666667	99.77272727	99.71130714
SVM	unit a	93.33333	93.61455903	93.19947321	93.02379196
	unit b	87.77778	87.25926801	87.80801963	87.07623522
	unit c	97.5	97.84383073	96.88950246	97.2093589
	unit d	90.83333	90.48053847	90.82338957	90.31944654
	unit e	88.05556	87.73899004	87.79290391	87.43322891
	unit f	87.77778	87.61287974	87.27652883	87.03883837
	SD-SERS arrays	100	100	100	100

Supplementary References:

1 Kresse, G. & Hafner, J. Ab initio molecular-dynamics simulation of the liquid-metal–amorphous-semiconductor transition in germanium. *Physical Review B* **49**, 14251 (1994).

2 Kresse, G. & Furthmüller, J. Efficient iterative schemes for ab initio total-energy calculations using a plane-wave basis set. *Physical review B* **54**, 11169 (1996).

3 Blöchl, P. E. Projector augmented-wave method. *Physical review B* **50**, 17953 (1994).

4 Kresse, G. & Joubert, D. From ultrasoft pseudopotentials to the projector augmented-wave method. *Physical review b* **59**, 1758 (1999).

5 Hammer, B., Hansen, L. B. & Nørskov, J. K. Improved adsorption energetics within density-functional theory using revised Perdew-Burke-Ernzerhof functionals. *Physical review B* **59**, 7413 (1999).

6 Monkhorst, H. J. & Pack, J. D. Special points for Brillouin-zone integrations. *Physical review B* **13**, 5188 (1976).

7 Gupta, P. *et al.* On-demand electromagnetic hotspot generation in surface-enhanced Raman scattering substrates via “add-on” plasmonic patch. *ACS applied materials & interfaces* **11**, 37939-37946 (2019).

8 Östmark, H., Wallin, S. & Ang, H. G. Vapor pressure of explosives: a critical review. *Propellants, Explosives, Pyrotechnics* **37**, 12-23 (2012).

9 Wang, J. *et al.* Inkjet-printed silver nanoparticle paper detects airborne species from crystalline explosives and their ultratrace residues in open environment. *Anal. Chem.* **86**, 3338-3345 (2014).

10 Gershanik, A. P. & Zeiri, Y. Sublimation rate of TNT microcrystals in air. *The Journal of Physical Chemistry A* **114**, 12403-12410 (2010).

11 McKone, T. E. & Daniels, J. I. Estimating human exposure through multiple pathways from air, water, and soil. *Regul. Toxicol. Pharm.* **13**, 36-61 (1991).
