# Supplementary material for: SERS nose arrays based on a signal differentiation approach for TNT gas detection
Source: Commun Chem. 2025 Aug 18;8:250. doi: 10.1038/s42004-025-01656-2 (PMC12361517; doi:10.1038/s42004-025-01656-2)
Supplement: Supplementary file 3 — Description of Additional Supplementary Files [file 42004_2025_1656_MOESM3_ESM.pdf]

Description of Additional Supplementary Files

File name- Supplementary Data 1

File description - The original data displayed in the main manuscript are provided in Supplementary Data 1.

File name- Supplementary Data 2

File description – Dataset of Supplementary Information is provided in Supplementary Data 2
